# Persistence of *Escherichia coli* O157:H7 and Its Mutants in Soils

**DOI:** 10.1371/journal.pone.0023191

**Published:** 2011-08-03

**Authors:** Jincai Ma, A. Mark Ibekwe, Xuan Yi, Haizhen Wang, Akihiro Yamazaki, David E. Crowley, Ching-Hong Yang

**Affiliations:** 1 United States Salinity Laboratory, Agriculture Research Service, United States Department of Agriculture, Riverside, California, United States of America; 2 Department of Environmental Sciences, University of California Riverside, Riverside, California, United States of America; 3 Department of Biological Sciences, University of Wisconsin, Milwaukee, Wisconsin, United States of America; 4 Institute of Soil and Water Resources and Environmental Science, Zhejiang University, Hangzhou, China; 5 Zhejiang Provincial Key Laboratory of Subtropical Soil and Plant Nutrition, Zhejiang University, Hangzhou, China; Institut de Pharmacologie et de Biologie Structurale, France

## Abstract

The persistence of Shiga toxin-producing *E. coli* O157:H7 in the environment poses a serious threat to public health. However, the role of Shiga toxins and other virulence factors in the survival of *E. coli* O157:H7 is poorly defined. The aim of this study was to determine if the virulence factors, *stx*_1_, *stx*_2_, *stx*_1–2_, and *eae* in *E. coli* O157:H7 EDL933 play any significant role in the growth of this pathogen in rich media and in soils. Isogenic deletion mutants that were missing one of four virulence factors, *stx*_1_, *stx*_2_, *stx*_1–2_, and *eae* in *E. coli* O157:H7 EDL933 were constructed, and their growth in rich media and survival in soils with distinct texture and chemistry were characterized. The survival data were successfully analyzed using Double Weibull model, and the modeling parameters of the mutant strains were not significantly different from those of the wild type. The calculated *T_d_* (time needed to reach the detection limit, 100 CFU/g soil) for loamy sand, sandy loam, and silty clay was 32, 80, and 110 days, respectively. It was also found that *T_d_* was positively correlated with soil structure (*e.g.* clay content), and soil chemistry (*e.g.* total nitrogen, total carbon, and water extractable organic carbon). The results of this study showed that the possession of Shiga toxins and intimin in *E. coli* O157:H7 might not play any important role in its survival in soils. The double deletion mutant of *E. coli* O157:H7 (*stx*_1_^−^ *stx*_2_^−^) may be a good substitute to use for the investigation of transport, fate, and survival of *E. coli* O157:H7 in the environment where the use of pathogenic strains are prohibited by law since the mutants showed the same characteristics in both culture media and environmental samples.

## Introduction

*Escherichia coli* O157: H7 was initially identified as an important human pathogen in 1982 during an investigation into a food-borne disease outbreak in the United States [1]. Since then, an increasing number of *E. coli* O157:H7 outbreaks have been reported in the United States. It is estimated that in the United States *E. coli* O157: H7 alone is responsible for a total of 73,480 cases of disease per year, among which, there are more than 1,800 cases of hospitalizations and 52 deaths. Evidence has shown that *E. coli* O157:H7 is one of the most commonly isolated bacterial pathogens from meat and fresh produce after *Campylobacter*, *Salmonella*, and *Shigella* spp [2]. In addition to the USA, many large outbreaks of *E. coli* O157:H7 infections have also been reported in many countries making *E. coli* O157:H7 an increasing public health concern worldwide. The infectious threshold of *E. coli* O157:H7 is very low, and ingestion of 10 cells may be enough to cause severe gastrointestinal illness [3]. The typical clinical symptoms of *E. coli* O157:H7 infections are watery diarrhea and hemorrhagic colitis [1], which can progressively develop into life-threatening hemolytic uremic syndrome (HUS) [4], [5].

Outbreaks of *E. coli* O157: H7 infections are always traced back to consumption of food that has been directly or indirectly contaminated by manure/water containing *E. coli* O157:H7. Animals including deer, horses, dogs, and birds [6], [7], [8], [9] are known to be *E. coli* O157:H7 carriers. However, cattle are thought to be the main carrier of *E. coli* O157:H7 [10], [11]. *E. coli* O157:H7 in the environment may originate from farms where manure amendments are used as fertilizer. The pathogen could be mobilized through irrigation water, providing an opportunity for the pathogen to spread out into its secondary reservoir, typically water and soil. The persistence and regrowth in these habitats may increase the potential for the pathogen to enter into the food chain and thereby constitute a public health risk. There have been some cases of infection from direct contact with *E. coli* O157:H7 contaminated soil, and more cases of food poisoning caused by or consumption of vegetables grown in soils contaminated by *E. coli* O157:H7 [12], [13].

The survival of *E. coli* O157:H7 in water [14], [15], [16], [17], [18], [19], manure and manure slurry [20], [21], [22], manure-amended soil [23], [24], [25], and sediment [26], [27], is well documented with sporadic reports in natural soils [28], [29]. More direct results could be obtained by applying pathogenic strain in the survival experiments [30], however, most of the studies used nonpathogenic *E. coli* O157:H7 strains [22], [24], [31], [32] due to environmental safety and regulations. This raises the question on how well those indirect results can represent results using pathogenic strains for comparison. Therefore, additional evidence is needed to clearly understand the role of *stx* genes and other virulence factors in the survival of pathogenic *E. coli* O157:H7 in the environment. Previous work [20] showed that there was a similar survival pattern between a Shiga toxin negative *E. coli* O157:H7 strain and a Shiga toxin positive *E. coli* O157:H7 strain. However, these strains were not isogenic, and the minor differences in survival might be attributed to other factors, such as the differences in their genomic DNA. Indeed, the variability in growth and survival of *E. coli* in soils has been shown to be strain-dependent [28].

**Figure 1:**
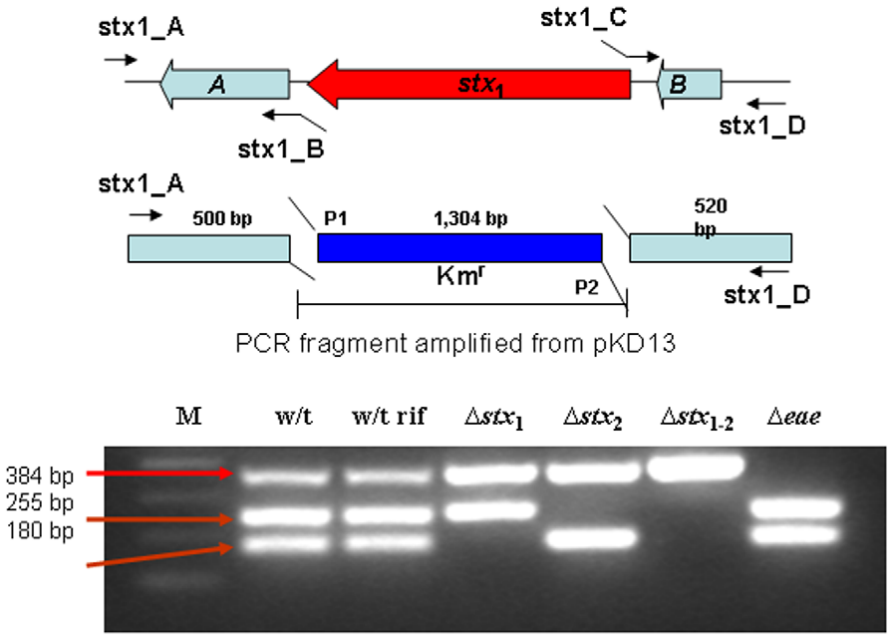
Construction of *stx* and *eae* mutants (top) and multiplex PCR confirmation of the mutant constructed (bottom). M represents 100 bp λDNA ladder.

In the current study, we chose *E. coli* O157:H7 EDL933 as the model pathogenic *E. coli* since its genome has been fully sequenced and annotated [42]. *E. coli* O157:H7 EDL933 and its isogenic mutant derivatives that are missing one of the following virulence factors, *stx*_1_, *stx*_2_, *stx*_1–2_, and *eae*, were constructed, their growth in rich medium and survival in soils compared to that of the wild type parental strain (Fig. 1). We hypothesized that since all of the strains are isogenic the results will provide insights into the role of *stx* and *eae* genes in the survival of *E. coli* O157:H7 in soils. Additionally, the survival of the *E. coli* O157:H7 EDL933 in soils will correlate with the survival of pathogenic *E. coli* O157:H7 strains in the environment.

## Materials and Methods

### Bacterial strains, construction and growth of mutants

The bacteria and plasmids used in this study are listed in Table 1. In order to facilitate the enumeration of *E. coli* O157:H7 EDL933 on selective media, the *E. coli* O157:H7 wild type was tagged with nalidixic acid in addition to rifampicin resistance, and its growth curve in LB (Luria-Bertani) broth was found to be identical to that of the non-tagged wild-type strain.

**Table 1:** Bacterial strains and plasmids.

Strain or plasmid	Relevant characteristics	Source or reference
strains		
*E. coli* DH5α	General laboratory strain	Gibco-BR
*E. coli* S17-1	General laboratory strain	Simon *et al.* 1983
*E. coli* EDL933	wild type	ATCC 43895
*E. coli* EDL933	rifampicin tagged, Rif^r^	This study
*E. coli* EDL933	*stx*1(del), , Km, Km^r^	This study
*E. coli* EDL933	*stx*2(del), , Cm, Cm^r^	This study
*E. coli* EDL933	*stx*1–2(del), , KmCm, Km^r^Cm^r^	This study
*E. coli* EDL933	*eae*(del), , Km, Km^r^	This study
plasmids		
pWM91	Suicide vector, Ap^r^	Metcalf *et al.*, 1996
pKD3	plasmid carrying Cm resistance cassette, Cm^r^	Datsenko and Wanner, 2000
pKD4	plasmid carrying Km resistance cassette, Km^r^	Datsenko and Wanner, 2000

Rif^r^, rifampicin resistance; Km^r^, kanamycin resistance; Cm^r^, chloramphenicol; Ap^r^, ampicillin resistance.

Mutants lacking Stx_1_, Stx_2_, and Eae were generated by allelic exchange protocol [33]. The flanking regions were amplified by PCR with specific primers (Table 1), among which primers B and C (e.g. stx1_B, stx1_C) have the linkers at the 5′ end that are complimentary to primers P1 and P2 [14], respectively, for crossover PCR. The kanamycin (Km) cassette was amplified from pKD4 (GenBank accession #, AY048743.1) and the chloramphenicol (Cm) cassette was amplified from pKD3 (GenBank accession #, AY048742.1) using the universal primer set consisting of forward primer P1 and reverse primer P2. Three-way crossover PCR was performed using the flanking regions and Km or Cm cassette as templates, and primers A and D (e.g. stx1_A, stx1_D) were used in this process. The PCR product was then cloned into pWM91 digested with *Xcm*I (T-vector). The resulting plasmid was transformed into *E. coli* S17-1 λ *pir*, and then introduced into EDL933 by transconjugation. Recombinants resulting from double crossover events were obtained by *sacB* and sucrose positive selection. All the mutant strains and the wild type strain were separately stored under −80°C on cryoprotective beads in MicroBank microbial storage tubes (Pro-Lab Diagnostics, Ontario, Canada).

The *stx* and *eae* mutants, together with the wild type strain were inoculated into 100 ml of LB broth, and grew under 37°C with a rotation rate of 250 rpm. The optical density at 610 nm (OD_610 nm_) was monitored using a VIS-UV spectrophotometer (Pharmacia Biotech Inc. NJ). The OD_610 nm_ was plotted against incubation time, and the apparent growth rate (*k*, h^−1^) was calculated using the following equation,
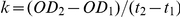
where *OD*_1_, *OD*_2_ are the optical density measured at time t_1_ and time t_2_, respectively, *k* is the apparent growth rate (h^−1^).

### Multiplex PCR confirmation of mutants

Multiplex PCR was performed on the mutants and the wild type to confirm the deletions of *stx*_1_, *stx*_2_, *stx*_1–2_, and *eae* genes in their genomes. PCR was performed using Ready-to-Go PCR beads with the three primer pairs (Table 2) targeting *stx*_1_, *stx*_2_, and *eae* gene [34]. Thermocycler protocol included an initial denaturation at 95°C for 10 min, followed by 35 cycles of denaturation at 94°C for 30 s, annealing at 55°C for 30 s, and extension at 72°C for 40 s, and a final extension at 72°C for 5 min. The PCR product was resolved by electrophoresis on a 1.0% agarose gel. The gel was then stained with ethidium bromide, visualized and photographed using a gel imaging system (Bio-Rad Lab., Irvine, CA). The PCR products with the correct sizes were cloned into TOPO TA cloning kit (Invitrogen, Carlsbad, CA) according to manufacture's protocol, and the resulting plasmids were sequenced. DNA sequence analysis was performed using DNAStar software (Lasergene, Madison, WI). Database searches were conducted with identified open reading frames (ORFs) by using the BLAST algorithm (http://blast.ncbi.nlm.nih.gov) to confirm the deletion of the corresponding gene(s).

**Table 2:** Primers for mutants' construction and multiplex PCR.

Primers ID	Nucleotide sequence (5′ end to 3′ end)	Predicted product size (bp)	Source or reference
stx1_A	GGGTCCGGACGGTCATATGT	827	This study
stx1_B	gaagcagctccagcctacacTCAGTGAAAATAGCAGGCGC		
stx1_C	ctaaggaggatattcatatGACCCCCTGAAGGACGGCGTTTT	814	This study
stx1_D	CACCCATTGCCGCCGGATTT		
stx2_A	CATGCTGATGATGCTGGGAGTG	781	This study
stx2_B	gaagcagctccagcctacacGCGCGTTGTACTGGATTCGA		
stx2_C	ctaaggaggatattcatatGAACCTGATTCGTGGTATGTGGG	801	This study
stx2_D	TGGATCAGGGCTGTCGAATG		
eae_A	GCAATAACCAAATCATATCCGC	852	This study
eae_B	gaagcagctccagcctacacAACCACCCCGGCTAAAATATGT		
eae_C	ctaaggaggatattcatatGCTCGAGTTTTTCAGGGGTAGCA	799	This study
eae_D	TCCAGCATAGGGACCGTGCA		
P1	GTGTAGGCTGGAGCTGCTTC	1463 or 1014	Datsenko and Wanner, 2000
P2	CATATGAATATCCTCCTTAGTTCC		
stx1_F	ATAAATCGCCATTCGTTGACTAC	180	Paton and Paton, 1998
stx1_R	AGAACGCCCACTGAGATCATC		
stx2_F	GGCACTGTACTGAAACTGCTCC	255	Paton and Paton, 1998
stx2_R	TCGCCAGTTATCTGACATTCTG		
eae_F	GACCCGGCACAAGCATAAGC	384	Paton and Paton, 1998
eae_R	CCACCTGCAGCAACAAGAGG		

### Collection, characterization, and inoculation of soils samples

Dello loamy sand, Arlington sandy loam, and Willow silty clay were collected from Santa Ana River bed, fallow field at the University of California-Riverside, and Mystic Lake dry bed, California, respectively (Table 3). Arlington sandy loam is a typical agricultural soil found in Riverside, CA, while the other two soils are typical soil types used for cattle production in eastern and western Riverside County, USA. Permit was obtained from the University of California Riverside to collect the Arlington sandy loam. The soil from the Mystic Lake dry bed has high clay content (71%), and the soil from Santa Ana River bed has high sand content (99%). The texture and chemistry of the three soils are listed in Table 3. Soil samples were collected, sieved (2 mm), put into plastic bags, and stored at 4°C in dark. Soil properties characterized included, clay, silt, and sand content, water content, water holding capacity (WHC), soil organic carbon (OC), and total nitrogen (T-N) [35]. Soil microbial biomass carbon (MBC) was extracted by the chloroform-fumigation-extraction method [36], and water extractable organic carbon (WEOC) was measured by a total organic carbon analyzer (TOC-500, Shimadzu Corp., Kyoto, Japan) according to the method by Liang et al. [37]. The assimilable organic carbon (AOC) fraction in WEOC was determined using a luminous bacterium strain, *Vibrio harveyi* (Ma et al., unpublished).

**Table 3:** Soil texture and chemistry.

Soil type	Sand (%)	Silt (%)	Clay (%)	Bulk density (g/cm)	WHC (%)	pH	T-N (g/kg)	OC (g/kg)	WEOC (mg/kg)	MBC ((mg/kg)	AOC ((mg/kg)
Dello loamy sand	99.1	0.2	0.7	1.67	17	7.1	0.07	0.58	10	11	0.20
Arlington sandy loam	70.9	20.8	8.3	1.54	21	7.2	0.61	5.40	44	56	0.90
Willow silty clay	3.7	49.1	47.2	1.51	63	7.2	1.61	20.4	242	278	4.94

WHC, water holding capacity; T-N, total nitrogen; OC, organic carbon; MBC, microbial biomass carbon; AOC, assimilable organic carbon.

One cryoprotective bead from MicroBank microbial storage tube containing *E. coli* O157:H7 was aseptically transferred to a 15 ml tube containing 5.0 ml LB broth and incubated at 37°C for 18 h. From the overnight culture, a 1.0 ml aliquot was transferred into a 250 ml flask containing 100 ml LB broth, and incubated at 37°C for 18 h to achieve early stationary phase. Stationary phase cells were used because in the natural environment, the majority of bacteria exist in this condition [38]. The cells were harvested by centrifugation at 3500 g (Beckman, Brea, CA), washed three time using phosphate buffer (10 mM, pH 7.2), and finally resuspended in sterile deionized water. The wash step was essential to remove the nutrient, typically organic carbon from the LB broth, since *E. coli* O157 is able to grow at low carbon concentrations in freshwater [39].

Cell from stock cultures were streaked on LB agar (without antibiotics), and incubated 37°C overnight. Single colonies were picked and restreaked onto LB agar with appropriate antibiotics. Single colonies were streaked onto SMAC (sorbitol MacConkey) agar supplemented with BCIG (5-bromo-4-chloro-3-indoxyl-ß-D-glucuronide) (Lab M, Lancashire, UK). The isolated colonies were inoculated into 100 ml LB broth with appropriate antibiotics (Table 1), and incubated at 37°C for about 16 h. The overnight culture were harvested by centrifugation at 4°C, washed three times with phosphate buffer (10 mM, pH 7.2), resuspended in sterile deionized water, and inoculated into soil samples. Cell concentrations in soils were about 0.5×10^7^ CFU per gram soil (g/dw) according to Franz et al. [22]. Briefly, the cell suspension was thoroughly mixed with soil in a plastic bag and 500 gram of the inoculated soil was transferred to a top perforated plastic bag for air exchange. The same amount of non-inoculated soil was put into another plastic bag, which was used as uninoculated control, with deionized water added instead of cell suspension. The experiment use triplicate bags of soils. The plastic bags were weighed and incubated at 10°C in darkness. Moisture content of the soil sample were adjusted to 60% water holding capacity (WHC), and water concentration was maintained during the course of experiment by adding additional deionized water weekly to obtain the original weight. Antibiotics were added into the agar media at the following concentrations, kanamycin (Km), 50 µg/ml; chloramphenicol (Cm), 25 µg/ml; rifampicin (Rif), 100 µg/ml; and nalidixic acid (Nal), 25 µg/ml.

### Sampling and enumeration

The inoculated soils were sampled periodically to determine the survival of the wild-type and mutant strains over time. At each point, two samples (1.0 g) of each triplicate bag was removed from the middle of the soil sample and put into pre-weighed dilution tubes. The tubes containing soil samples were weighted to calculate the exact size of soil sample. A 5.0 ml of 0.1% peptone buffer (Lab M, Lancashire, UK) was added to the test tube containing the soil sample, and the soil was thoroughly mixed with the buffer by inverting the tube several times and then vortexed for 2×20 s. The resulting soil paste (cell suspension) was then subjected to 10-fold serial dilutions. Fifty µl of the two highest dilutions were plated in duplicate on SMAC/BCIG agar with appropriate antibiotics for enumeration. The inoculated SMAC agar plates were incubated at 37°C for 16 h, and the results expressed as log colony forming units per gram dry weight (CFU g/dw). The detection limit of the plating method was approximately 100 CFU g/dw. Our preliminary experiments showed that the average cell recovery rate of the method was from 90 to 110% of the theoretical value.

### Survival data

Survival of *E. coli* O157:H7 was modeled by fitting the experimental data to the double Weibull survival model proposed by Coroller et al. [40] using GInaFiT version 1.5 developed by Dr. Annemie Geeraerd at Katholieke Universiteit, Leuven, Belgium [41]. The double Weibull survival model was constructed based on the hypothesis that the population is composed of two subpopulations differing in their capability on resistance to stress, and deactivation kinetics of both subpopulations follows a Weibull distribution. The size of the surviving population can be calculated using equation 1,
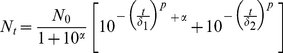
(1)

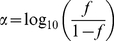
(2)Where *N* is the number of survivors, *N*_0_ is the inoculum size; *t* is the time; *p* is the shape parameter, when *p*>1 a convex curve is observed; when *p*<1 a concave curve is observed, when *p* = 1 a linear curve is observed. The scale parameter, *δ*, represents the time needed for first decimal reduction; *f*, varying from 0 to 1, is the fraction of subpopulation 1 in the population. Another parameter, α, varying from negative infinity to positive infinity, is obtained by logit transformation of *f* as shown in equation 2. The strong correlation between the scale (*δ*) and the shape (*p*) parameters makes the double Weibull model to fit most of the shapes of deactivation curves. Previous study proved that the double Weibull model can successfully describe a biphasic shape with nonlinear decrease, which can not be described by other survival models [40]. Additionally, when *δ*_1_ = *δ*_2_, the double Weibull model can be simplified into a single Weibull model, and the survival curve can be described by only three parameters. A very important and useful parameter, Td (time needed to reach detection limit, 100 CFU g/dw) can also be calculated when using GInaFiT to fit the experimental survival data.

### Statistical analysis

Analysis of variance (ANOVA) was performed to investigate the differences in growth in rich medium, and the survival in soils using SPSS 16.0 software package (Chicago, IL).

## Results

### Mutant construction and confirmation

The genome of *E. coli* O157:H7 EDL933 has been fully sequenced and annotated [42], which makes it possible to knock out the genes of interest. The multiplex PCR assay (Fig. 1) clearly showed that the wild type strains displayed three bands representing the amplicons from *eae*, *stx*_2_, and *stx*_1_ genes, from top to bottom, with predicted sizes of 384, 255, and 180 bp, respectively. For the mutant derivatives, there was one band missing for Δ*stx*_1_, Δ*stx_2_*, and *Δeae*, and two bands missing for the double mutant construct *Δstx*_1–2_, compare to the wild type strain. Figure 1 clearly showed that the virulence factors were successfully deleted as evidenced by the missing of the corresponding bands on the agarose gel.

### Growth in rich medium and survival in soils

**Figure 2:**
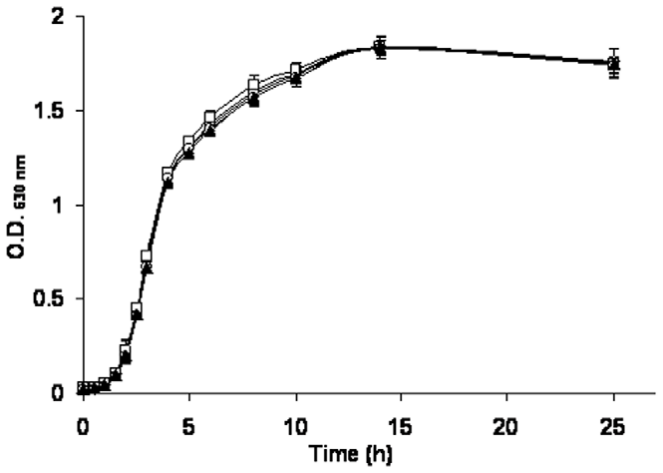
Growth curves of wild type strain (•) and its derivative mutants strains, Δ*stx*_1_ (◊), Δ*eae* (▴), Δ*stx*_2_ (□), and Δ*stx*_1–2_ (○). The data represent the average of triplicate measurements.

The growths of the mutant strains in LB broth were compared with that of the wild type (Fig. 2). The results showed that the *E. coli* O157:H7 EDL933 mutant derivatives growth was not significantly different from that of the wild type. Overall, there was about 1.5 h lag time followed by an exponential phase (5 h from incubation), then stationary phase (8 h post inoculation), and no decay phase was observed until 25 h of growth in LB broth under 37°C. The calculated apparent growth rates (*r*) of wild type, Δ*stx*_1_, Δ*eae*, Δ*stx*_2_, and Δ*stx*_1–2_ were as following, 0.46±0.02, 0.46±0.03, 0.46±0.03, 0.47±0.04, and 0.46±0.03.

**Figure 3:**
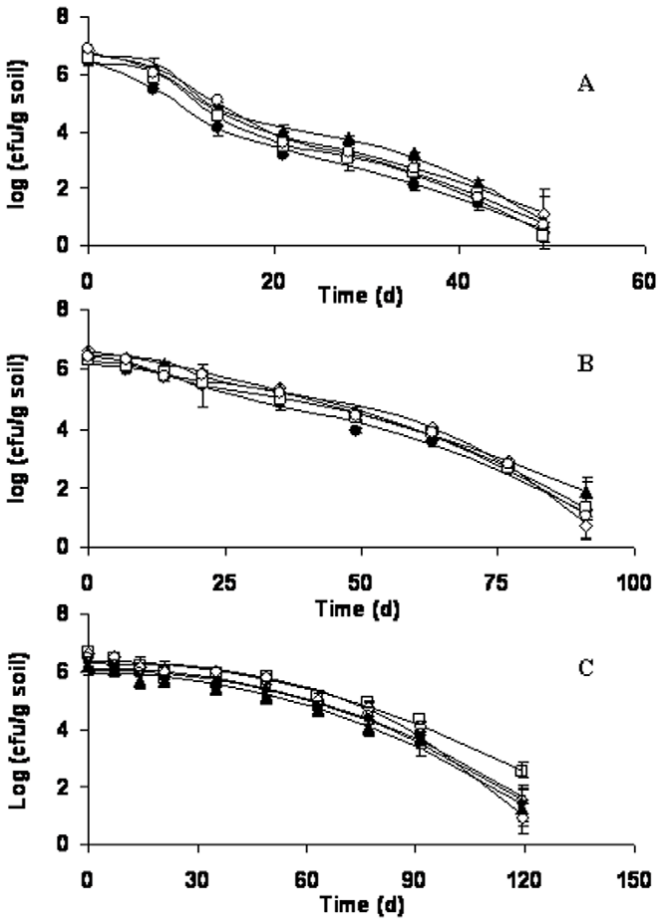
Survival of the wild type (•) and its mutant derivatives, Δ*stx*_1_ (◊), Δ*eae* (▴), Δ*stx*_2_ (□), and Δ*stx*_1–2_ (○), in loamy sand (3A), sandy loam (3B), and silty clay (3C). The data represent the average of triplicate experiments.

The wild type strain and the mutant strains were inoculated in soils to test their survival at 10°C. The results (Fig. 3) showed that within the same soil, there were no significant differences in deactivation profiles between the mutant and the wild type strains. It was also observed that the survival varies greatly in different soils. The cells survived shortest (32 day) in loamy sand with less nutrients (Fig. 3A), longest survival (113 day) was found in silty clay soil where there are more finer particles and more nutrient (e.g., organic carbon, nitrogen) (Fig. 3C), while the survival length was intermediate (82 day) in sandy loam soil. In loamy sand (Fig. 3A), there was a sharp decline of cell population within the first two weeks post inoculation, followed by a steady decrease until cell concentration dropped below detection limit. In sandy loam (Fig. 3B), a similar trend was also observed, a quick drop during first two weeks followed by a progressive decline. While in the silty clay (Fig. 3C), cells survived longer, because the cell concentrations did not decline significantly until four weeks post inoculation. Here after, cells started a very slow decline and dropped below detection limit (100 CFU g dw^−1^) after 113 days.

### Modeling of survival data

**Figure 4:**
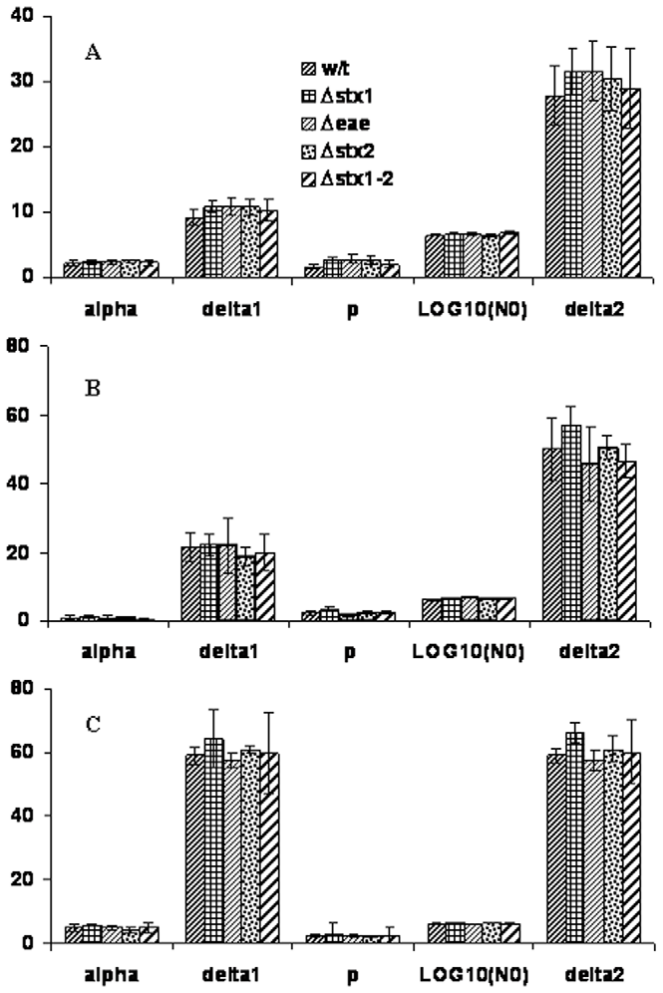
Double Weibull Model parameters of wild type strain and its mutant derivatives in loamy sand (4A), sandy loam (4B), and silty clay (4C).

To accurately compare the survival kinetics between the wild type and mutant strains, survival data were modeled using a double Weibull equation as shown in Fig. 4. Similar modeling parameters (*α*, *δ*, and *p*) from mutant strains and the wild type strain were calculated when they were inoculated into the same soil. However, more variations in these parameters were observed from different soils, especially the *δ* values. When these strains were characterized in loamy sand and sandy loam soils, distinct *δ*_1_ and *δ*_2_ were observed indicating that the two subpopulations behave differently in both soils. The subpopulation with greater *δ* value declines slower than the one with smaller *δ* value. In contrast, almost identical *δ*_1_ and *δ*_2_ values were calculated from the survival data in silty clay soil indicating that the two subpopulations of cells in this soil likely behave similarly, thus the survival data in silty clay might be simplified into one Weibull model that can be described by only three parameters, α, *δ* and *p*. The initial sharp decrease in cell numbers in loamy sand soil might largely be attributed to the faster decline of subpopulation with smaller *δ*. However, with the time, the subpopulation with greater *δ* dominated the cell population, leading to a slower and steadier decline of the cell concentrations. A similar trend was also observed in sandy loam soil. However, in silty clay soil, the cell concentrations did not change until 3 weeks post inoculation. This was followed by a steady decrease in cell concentrations until the population dropped below the detection limit (57–66 days).

**Figure 5:**
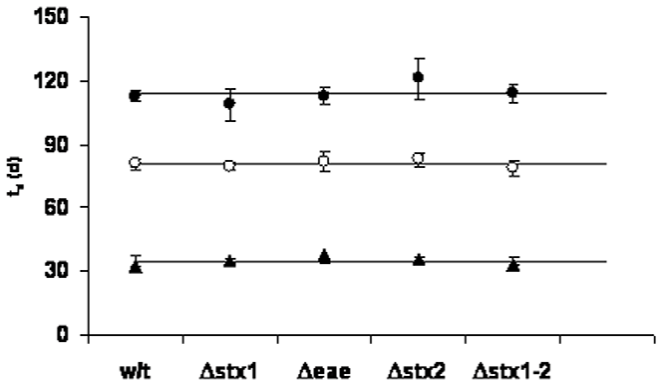
*Td* values calculated from the double Weibull model for wild type and its mutants derivatives in loamy sand (▴), sandy loam (○), and silty clay (•). The data represent the average of triplicate modeling of the raw survival data.

**Figure 6:**
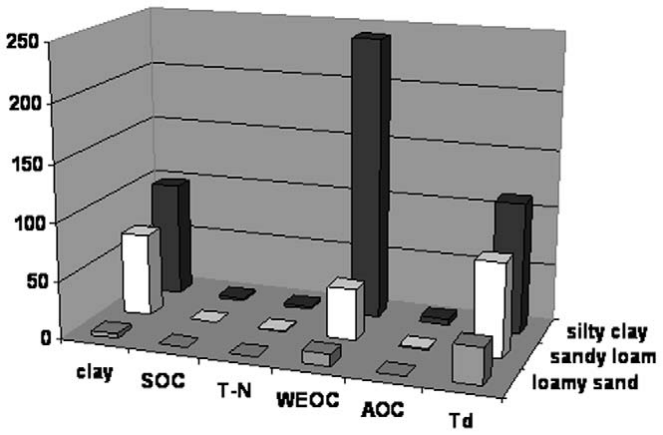
Effects of clay content (%), soil organic carbon (OC, %), total nitrogen (T-N, %), water extractable organic carbon (WEOC, mg/kg) *Td*. Gray, white, and black columns represent loamy sand, sandy loam, and silty clay, respectively.

The time to reach detection limit (*Td*) between the wild type and mutant strains in the same soil was not significantly different (*P* = 0.05) (Fig. 5). *Td* values in soils follow the order of, silty clay>sandy loam>loamy sand, which is consistent with the order of fine particle and nutrient levels in the soils. The effect of soil properties on the survival of *E. coli* O157:H7, and the time that it takes for the pathogen to reach detection limit was determined (Fig. 6). The results showed that with the increase in clay content, total organic carbon, total nitrogen, and water extractable organic carbon, there was a corresponding increase in *Td* values.

## Discussion

The most significant finding of this work is Shiga toxins and intimin have no influence on the survival of pathogenic *E. coli* O157:H7 EDL933 in the three soils. The Shiga toxins *stx*_1_, *stx*_2_ genes, and *eae* gene in *E. coli* O157:H7 have been intensively investigated [43], [44]. Shiga toxins might induce an advantage in *E. coli* O157:H7 survival in the environment [44], [45], [46]. However, the role of these genes in survival of the pathogenic *E. coli* O157:H7 is still not completely understood [20]. Most of the previous survival studies used nonpathogenic *E. coli* O157:H7 strains [30], [24], [31], [32], and the survival data based on pathogenic strains in the environment are not available due to regulations and safety concerns [27], [28]. The typical nonpathogenic *E. coli* O157:H7 strain widely used in the literature include a green fluorescence protein labeled strain (*E. coli* O157:H7 B6-914 GFP-91) constructed by Fratamico et al. [47] and a bioluminescent construct (*E. coli* O157:H7 Tn*5*, *luxCDABE*) by Ritchie et al. [31], both of which have been shown to survive in soil for months. Researchers used these nonpathogenic strains in the survivals studies on the assumption that they behave the same with the pathogenic strains. Therefore, comparative studies relating the survival of pathogenic *E. coli* O157:H7 strains to nonvirulent strains are needed to make a firm conclusion. The study by Kudva and colleagues [7] revealed that identical or very similar survival patterns were observed within a Shiga toxin positive *E. coli* O157:H7 strain (ATCC 43894) and Shiga toxin negative *E. coli* O157:H7 strain (ATCC 43888), indicating that Shiga toxins might have little or no influence on *E. coli* O157:H7 survival in manure and manure slurry. However, the strains used in their analysis were not isogenic, and factors other than Shiga toxins may have contributed to the minor survival differences observed in that study. In the present study, we have constructed a cluster of mutant derivatives from *E. coli* O157:H7 EDL933, with one of the following virulence factors deleted, *stx*_1_, *stx*_2_, *stx*_1–2_, and *eae*. The indistinguishable growth curves between the mutants and the wild type strains in rich medium, in combination with their similar survival profiles in three different soils, offer strong evidence that the Shiga toxin genes and *eae* gene do not likely play important role in the survival of *E. coli* O157:H7 in soils.

In the current study, the survival data were successfully modeled by the double Weibull model. Different models were fitted into the survival data, but the best fit was obtained by applying double Weibull model. Since double Weibull model was based on the assumption that there are two subpopulations, and they differs in level of resistance to stress, and the survival of both subpopulations follow a Weibull distribution. Subpopulation with smaller δ die off faster compared to the other subpopulation with greater δ. In loamy sand, and sandy loam soils, distinct δ values, *i.e.* δ_1_≠δ_2_, were obtained for the two subpopulations, indicating that the two subpopulation exhibit different resistant capability in both soils. On the other hand, almost identical δ values, *i.e.* δ_1_≈δ_2_, were observed for the two subpopulations in silty clay soil, implying that the two subpopulations show a similar survival behavior in silty clay soil.

The persistence of *E. coli* O157:H7 is highly dependent on soil types, since distinct persistence time (*Td*) of this pathogen varies significantly in different soils in terms of soil chemistry and texture. The longest survival was observed in silty clay soil, while the shortest survival was found in loamy sand soil. The results of soil characterization revealed that the silty clay soil is most abundant in clay, organic carbon, total nitrogen, and water extractable organic carbon, while the least abundant of those fractions is found in loamy sand soil. The variation in *Td* was best explained by the clay content in soils, since *Td* was closely correlated with the clay content. This agrees with the fact that the pathogens survived longer in finer-textured (clayey) than in courser (sandy) soils under similar environmental conditions [48]. Colonization of soil particles and aggregates is thought to be critical for the inoculated bacteria to survive in soil [49]. Finer textured soils (clayey) compared to coarser textured soils (sandy) may provide protective pore spaces to improve the survival of soil bacteria [50]. Indeed, the survival of a bacterial pathogen in 23 soils types was found to be positively correlated with soil clay content, in addition to other factors [51]. Indeed, greater survival of *E. coli* in sediment rich in clay (>25%) has been observed [25].Similarly, survival of *E. coli* O157:H7 was primarily determined by the soil texture, with prolonged survival associated with more clay particles compared with sand particles [22], [52], [53]. In addition to soil texture, soil chemistry characteristics, such as organic carbon, total nitrogen, and water extractable organic carbon, were also found to be positively related to survival of *E. coli* O157:H7. In our study, the availability of nutrient, such as nitrogen and organic carbon in soil were found to correlate with the pathogen survival in soils. Recently, Franz et al. [22] showed that the survival of *E. coli* O157 in 36 soils can best be explained by dissolved organic carbon and the ratio of dissolved organic carbon to microbial biomass carbon. In addition to soil texture and soil chemistry, biological factors cannot be neglected when interpreting the survival data of *E. coli* O157:H7 in soils. Overall, soils that are rich in clay or organic carbon might be a good secondary medium for extended persistence of *E. coli* O157:H7. Special attention should be paid to such soils when evaluating the environmental risk associated with *E. coli* O157:H7. The studies by the above authors and a recent review [54], to the best of our knowledge, have produced the most up to date data on survival of *E. coli* O157:H7 in soil. The review showed that temperature, soil structure, and microbial communities are the most important factors affecting survival. These authors showed from their previous studies [32] that the survival of *E. coli* O157:H7 was inversely proportional to the diversity of the microbial community established through differential fumigation and regrowth activities. Niche dependency strategy has also been suggested as a mechanism for *E. coli* O157:H7 survival in the open environment [55] rather than the biphasic growth model tested in this study. This argument is based on nutrient availability as the most important physiological factor for survival of *E. coli* O157 in nutrient-limited environment. However, we did not test this phenomenon in this study, but further studies in our laboratory will be looking at this in the nearest future.

In summary, *stx*_1_, *stx*_2_, and *eae* genes conferred in *E. coli* O157:H7 EDL933 did not play any direct role in survival of this pathogen in soil because the isogenic mutant strains showed indistinguishable survival profiles in three soils with distinct soil chemistry. The survival results obtained based on the non-pathogenic isogenic *E. coli* O157 strains from this study might be safely extrapolated to be equivalent to data obtained from pathogenic strains since the survival data from pathogenic strains in the environment are not available due to regulations and safety concerns. However, other conditions should be considered, *e.g.*, genes other than *stx* and *eae* that might be important in *E. coli* O157 survival in the environment. Best management practices (BMPs) and good agricultural practices (GAPs) must be followed when leafy greens are grown in soils with high clay and organic carbon contents to reduce the risk of such soils being contaminated with *E. coli* O157:H7.

## References

[1] Riley L, Remis RS, Helgerson SD, McGee HB, Wells JG (1983). Hemorrhagic colitis associated with a rare *Escherichia coli* serotype.. N Engl J Med.

[2] Mead PS, Slutsker L, Dietz V, McCaig LF, Bresee JS (1999). Food-related illness and death in the United States.. Emerg Infect Dis.

[3] Griffin PM, Tauxe RV (1991). The epidemiology of infections caused by *Escherichia coli* O157:H7, other enterohemorrhagic *E. coli*, and the associated hemolytic uremic syndrome.. Epidemiol Rev.

[4] Carter AO, Borczyk AA, Carlson JA, Harvey B, Hockin JC (1987). A severe outbreak of *Escherichiacoli* O157:H7.associated hemorrhagic colitis in a nursing home.. N Engl J Med.

[5] Karmali MA, Petric M, Lim C, Fleming PC, Steele BT (1983). *Escherichia coli* cytotoxin, haemolytic–uraemic syndrome, and haemorrhagic colitis.. Lancet.

[6] Chapman PA, Siddons CA, Cerdan-Malo AT, Harkin MA (1997). A 1-year study of *Escherichia coli* O157 in cattle, sheep, pigs and poultry.. Epidemiol Infect.

[7] Kudva IT, Hatfield PG, Hovde CJ (1997). Characterisation of *Escherichia coli* O157:H7 and other shiga toxin-producing *E. coli* serotypes isolated from sheep.. J Clin Microbiol.

[8] Rice DH, Hancock DD, Besser TE (1995). Verotoxigenic *E. coli* colonisation of wild deer and range cattle.. Vet Rec.

[9] Wallace JS, Cheasty T, Jones K (1997). Isolation of verocytotoxin producing *Escherichia coli* O157 from wild birds.. J Appl Microbiol.

[10] Armstrong GL, Hollingsworth J, Morris JG (1996). Emerging foodborne pathogens, *Escherichia coli* O157:H7 as a model of entry of a new pathogen into the food supply of the developed world.. Epidemiol Rev.

[11] Chase-Topping M, Gally D, Low C, Mathews L, Woolhouse M (2008). Super-shedding and the link between human infection and livestock carriage of *Escherichia coli* O157.. Nature Rev Microbiol.

[12] Cieslak PR, Barrett TJ, Griffin PM, Gensheimer KF, Beckett G (1993). *Escherichia coli* O157:H7 infection from a manured garden.. Lancet.

[13] Morgan GM, Newman C, Palmer SR, Allen JB, Shepherd W (1988). First recognized community outbreak of haemorrhagic colitis due to verotoxin-producing *Escherichia coli* O157 in the UK.. Epidemiol Infect.

[14] Datsenko KA, Wanner BL (2000). One-step inactivation of chromosomal genes in *Escherichia coli* K-12 using PCR products.. Proc Natl Acad Sci U S A.

[15] Artz RRE, Killham K (2002). Surivival of *Escherichia coli* O157:H7 in private drinking water wells influences of protozoan grazing and elevated copper concentration.. FEMS Microbiol Lett.

[16] Avery LM, Williams AP, Killham K, Jones DL (2007). Survival of *Escherichia coli* O157:H7 in waters from lakes, rivers, puddles and animal-drinking troughs.. Sci Total Environ.

[17] McGee P, Bolton DJ, Sheridan JJ, Earley B, Kelly G (2002). Survival of *Escherichia coli* O157:H7 in farm water, its role as a vector in the transmission of the organism within herds.. J Appl Microbiol.

[18] Ravva SV, Sarreal CZ, Duffy B, Stanker LH (2006). Survival of *Escherichia coli* O157:H7 in wastewater from dairy lagoons.. J Appl Microbiol.

[19] Watterworth L, Rosa B, Schraft H, Topp E, Leung KT (2006). Survival of various ERIC-genotypes of Shiga toxin-producing *Escherichia coli* in well water.. Water, Air, Soil Pollut.

[20] Bolton DJ, Byrne CM, Sheridan JJ, McDowell DA, Blair IS (1999). The survival characteristics of a non-pathogenic strain of *Escherichia coli* O157:H7.. J Appl Microbiol.

[21] Kudva IT, Blanch K, Hovde CJ (1998). Analysis of *Escherichia coli* O157:H7 survival in ovine or bovine manure and manure slurry.. Appl Environ Microbiol.

[22] Williams AP, McGregor KA, Killham K, Jones DL (2008). Persistence and metabolic activity of *Escherichia coli* O157:H7 in farm animal faces.. FEMS Microbiol Lett.

[23] Franz E, Semenov AV, Termorshuizen AJ, de Vos OJ, Bokhorst JG (2008). Manure-amended soil characteristics affecting the survival of *E. coli* O157:H7 in 36 Dutch soils.. Environ Microbiol.

[24] Jiang X, Morgan J, Doyle MP (2002). Fate of *Escherichia coli* O157:H7 in Manure-Amended Soil.. Appl Environ Microbiol.

[25] Semenov A, Franz E, van Overbeek L, Termorshulizen AJ (2008). Estimating the stability of *Escherichia coli* O157:H7 survival in manure-amended with different management histories.. Environ Microbiol.

[26] Burton GA, Gunnison D, Lanza GR (1987). Survival of pathogenic bacteria in various freshwater sediments.. Appl Environ Microbiol.

[27] LaLiberte P, Grimess DJ (1982). Survival of *Escherichia coli* in lake bottom sediment.. Appl Environ Microbiol.

[28] Campbell GR, Prosser J, Glover A, Killham K (2001). Detection of *Escherichia coli* O157:H7 in soil and water using multiplex PCR.. J Appl Microbiol.

[29] Topp E, Welsh M, Tien Y-C, Dang A, Lazarovits G (2003). Strain-dependent variability in growth and survival of *Escherichia coli* in agricultural soil.. FEMS Microbiol Ecol.

[30] Vidovic S, Block HC, Korber DR (2007). Effect of soil composition, temperature, indigenous microflora, and environmental conditions on the survival of *Escherichia coli* O157:H7.. Can J Microbiol.

[31] Ritchie JM, Campbell GR, Shepherd J, Beaton Y, Jones D (2003). A Stable bioluminescent construct of *Escherichia coli* O157:H7 for hazard assessments of long-term survival in the environment.. Appl Environ Microbiol.

[32] van Elsas JD, Hill P, Chroňáková A, Grekova M, Topalova Y (2007). Survival of genetically marked *Escherichia coli* O157:H7 in soil as affected by soil microbial community shifts.. ISME J.

[33] Metcalf WW, Jiang WH, Daniels LL, Kim SK, Haldimann A (1996). Conditionally replicative and conjugative plasmids carrying *lac*Z alpha for cloning, mutagenesis, and allele replacement in bacteria.. Plasmid.

[34] Paton AW, Paton JC (1998). Detection and characterization of Shiga toxigenic *Escherichia coli* by using multiplex PCR assays for *stx*
_1_, *stx*
_2_, *eaeA*, enterohemorrhagic *E. coli hlyA*, *rfb*
_O111_, and *rfb*
_O157_.. J Clin Microbiol.

[35] Klute A (1996). Methods of soil analysis, part 1, physical and mineralogical methods (2nd edition), American Society of Agronomy, Agronomy Monographs 9(1), Madison, Wisconsin..

[36] Vance ED, Brookes PC, Jenkinson DS (1987). An extraction method for measuring soil microbial biomass C.. Soil Biol Biochem.

[37] Liang BC, MacKenzie AF, Schnitzer M, Monreal CM, Voroney PR (1998). Management-induced change in labile soil organic matter under continuous corn in eastern Canadian soils.. Biol Fertil Soils.

[38] Kolter R, Siegele DA, Tormo A (1993). The stationary phase of the bacterial cell.. Annu Rev Microbiol.

[39] Vital M, Hammes F, Egli T (2008). *Escherichia coli* O157 can grow in natural freshwater at low carbon concentrations.. Environ Microbiol.

[40] Coroller L, Leguerinel I, Mettler E, Savy N, Mafart P (2006). General model, based on two mixed Weibull distributions of bacterial resistance, for describing various shapes of inactivation curves.. Appl Environ Microbiol.

[41] Geeraerd AH, Valdramidis VP, van Impe JF (2005). GInaFiT, a freeware tool to assess non-log-linear microbial survivor curves.. Intl J Food Microbiol.

[42] Perna N, Plunkett G, Burland V, Mau B, Glasner JD (2001). Genome sequence of enterohaemorrhagic *Escherichia coli* O157:H7.. Nature.

[43] Besser RE, Griffin PM, Slustsker L (1999). *Escherichia coli* O157:H7 gastroenteritis and the hemolytic uremic syndrome: an emerging infection disease.. Annu Rev Med.

[44] Kaper JB, Nataro JP, Mobley HL (2004). Pathogenic *Escherichia coli*.. Nat Rev Microbiol.

[45] Lainhart W, Stolfa G, Koudelka GB (2009). Shiga Toxin as a bacterial defense against a eukaryotic predator, *Tetrahymena thermophila*.. J Bacteriol.

[46] O'Loughlin E, Robins-Browne RM (2001). Effect of Shiga toxins on eukaryotic cells.. Microbes Infect.

[47] Fratamico PM, Deng MY, Strobaugh TP, Palumbo SA (1997). Construction and characterization of *Escherichia coli* O157:H7 strains expressing firefly luciferase and Green fluorescent protein and their use in survival Studies.. J Food Prot.

[48] van Elsas JD, Dijkstra AF, Govaert JM, van Veen JA (1986). Survival of *Pseudomonas fluorescens* and *Bacillus subtilis* introduced into two soils of different texture in field microplots.. FEMS Microbiol Ecol.

[49] Hattori T, Hattori R (1976). The physical environment in soil microbiology, an attempt to extend principles of microbiology to soil microorganisms.. CRC Crit Rev Microbiol.

[50] van Veen JA, van Overbeek LS, van Elsas JD (1997). Fate and activity of microorganisms introduced into soil.. Microbiol Mol Biol Rev.

[51] Bashan Y (1986). Enhancement of wheat root colonization and plant development by *Azospirillum brasilense* Cd following temporary depression of rhizosphere microflora.. Appl Environ Microbiol.

[52] Mubiru DN, Coyne MS, Grove JH (2000). Mortality of *Escherichia coli* O157:H7 in two soils with different physical and chemical properties.. J Environ Qual.

[53] Nicholson FA, Groves SJ, Chambers BJ (2005). Pathogen survival during livestock manure storage and following land application.. Bioresource Technol.

[54] van Elsas JD, Semenov AV, Costa R, Trevors JT (2011). Survival of *Escherichia coli* in the environment: fundamental and public health aspects.. ISME J.

[55] Franz E, van Bruggen AHC (2008). Ecology of *E. coli* O157:H7 and Salmonella enterica in the primary vegetable production chain.. Crit Rev Microbiol.

